# The impact of behavioral interventions on co-infection dynamics: An exploration of the effects of home isolation

**DOI:** 10.1016/j.jtbi.2019.05.017

**Published:** 2019-09-07

**Authors:** Diana M Hendrickx, Steven Abrams, Niel Hens

**Affiliations:** a Center for Statistics, Interuniversity Institute for Biostatistics and statistical Bioinformatics, Hasselt University, Diepenbeek, Belgium; b Centre for Health Economics Research and Modelling Infectious Diseases, Vaccine and Infectious Disease Institute, University of Antwerp, Antwerp, Belgium

**Keywords:** Co-infection model, Partial differential equations, Reproduction numbers, Behavioral epidemiology

## Abstract

Behavioral epidemiology, the field aiming to determine the impact of individual behavior on the spread of an epidemic, has gained increased recognition during the last few decades. Behavioral changes due to the development of symptoms have been studied in mono-infections. However, in reality, multiple infections are circulating within the same time period and behavioral changes resulting from contraction of one of the diseases affect the dynamics of the other.

The present study aims at assessing the effect of home isolation on the joint dynamics of two infectious diseases, including co-infection, assuming that the two diseases do not confer cross-immunity. We use an age- and time- structured co-infection model based on partial differential equations. Social contact matrices, describing different mixing patterns of symptomatic and asymptomatic individuals are incorporated into the calculation of the age- and time-specific marginal forces of infection.

Two scenarios are simulated, assuming that one of the diseases has more severe symptoms than the other. In the first scenario, people stay only at home when having symptoms of the most severe disease. In the second scenario, twice as many people stay at home when having symptoms of the most severe disease than when having symptoms of the other disease.

The results show that the impact of home isolation on the joint dynamics of two infectious diseases depends on the epidemiological parameters and properties of the diseases (e.g., basic reproduction number, symptom severity). In case both diseases have a low to moderate basic reproduction number, and there is no home isolation for the less severe disease, the final size of the less severe disease increases with the proportion of symptomatic cases of the most severe disease staying at home, after an initial decrease. This counterintuitive result could be explained by a shift in the peak time of infection of the disease with the most severe symptoms, resulting in a smaller number of people with less contacts at the peak time of the other infection. When twice as many people stay at home when having symptoms of the most severe disease than when having symptoms of the other disease, increasing the proportion staying at home always reduces the final size of both diseases, and the number of co-infections.

In conclusion, when providing advise if people should stay at home in the context of two or more co-circulating diseases, one has to take into account epidemiological parameters and symptom severity.

## Highlights

• Home isolation impacts the dynamics of infection spread via close contacts.
• For multiple infections, the dynamic behaviour is interdependent.
• The interdependence depends on the epidemiological parameters and symptom severity.
• The effect on peak time and final size can be both positive and negative.

## 1 Introduction

Jointly modeling the dynamics of two or more infectious diseases with or without similar transmission routes can provide new insights in the interaction among these different pathogens (Hens, Aerts, Shkedy, Theeten, Van Damme, Beutels, 2008, Merler, Poletti, Ajelli, Caprile, Manfredi, 2008, Shrestha, Foxman, Weinberger, Steiner, Viboud, Rohani, 2013, Restif, Wolfe, Goebel, Bjornstad, Harvill, 2008). For airborne diseases, deterministic compartmental models described by ordinary differential equations (ODEs) have been proven to provide a suitable mathematical framework for studying such interactions (Merler, Poletti, Ajelli, Caprile, Manfredi, 2008, Restif, Wolfe, Goebel, Bjornstad, Harvill, 2008). Such ODE-based co-infection models typically describe the transmission dynamics of two (or more) infectious diseases, and the flow of individuals between different compartments or states (e.g., susceptible, infected, recovered), in function of calendar time. Alternatively, age-specific effects could be studied, at least when assuming endemic equilibrium for the infections at hand similarly (Hens, Shkedy, Aerts, Faes, Van Damme, Beutels, 2012, Rohani, Earn, Finkenstädt, Grenfell, 1998).

Apart from calendar time, age is also an important factor influencing the dynamics of infectious diseases. Within the same calendar year, transmission parameters can differ for people of various ages, e.g., for childhood diseases the infection risk tends to be lower for adults and elderly as compared to children. Hence, compartmental models including both calendar time and age effects provide a straightforward extension of the aforementioned models. The flow of individuals in such models is then described using a system of partial differential equations (PDEs) in time and age. Age structure can be included in the model via contact or mixing matrices, including social contact rates among individuals in different age categories, the population age distribution and age-specific mortality rates (Hens, Shkedy, Aerts, Faes, Van Damme, Beutels, 2012, Castillo-Chavez, Hethcote, Andreasen, Levin, Liu, 1989).

In addition to age and calendar time effects, implicitly decomposing the population in various subgroups, one can decompose the subpopulation of infectious individuals further into symptomatic and asymptomatic cases, which makes sense if for the pathogens under study the occurrence of asymptomatic infections is agreed upon. While most individuals change their social contact behavior when experiencing symptoms (Eames et al., 2010), at least when these symptoms are moderate to severe, by staying at home, asymptomatic individuals will show similar contact patterns as compared to individuals who are uninfected (either susceptible or immunized). Furthermore, symptomatic individuals are presumed to be more contagious than asymptomatic individuals, which has been demonstrated in the context of influenza by Van Kerckhove et al. (2013). Behavioral epidemiology has the aim to determine the effect of individual behavior on the spread of infectious diseases and has become a growing field during the last few decades (Manfredi and D’Onofrio, 2013). It’s increasingly recognized that human behavior affects the spread of infectious diseases, which has led to an increasing trend in incorporating human behavior into infectious disease modeling (Wang, Andrews, Wu, Wang, Bauch, 2015, Verelst, Willem, Beutels, 2016). Behavioral changes due to the development of symptoms and differences in contagiousness between symptomatic and asymptomatic people have recently been implemented and the effects thereof have been studied using compartmental models for mono-infections (Santermans et al., 2017). More specifically, using general practitioner data, these authors (Santermans et al., 2017) showed that in case of influenza, the total number of cases can be reduced by 39% or 63% when 50% or all symptomatic individuals, respectively, would stay at home immediately after the onset of symptoms.

The present study extends the work by Santermans et al. (2017) in the sense that our approach incorporates social contact matrices for both symptomatic and asymptomatic individuals, together with differences in infectiousness among those two groups, in an age- and time-structured co-infection model for two diseases which is described using a system of PDEs. We assume that there is no cross-immunity induced for the diseases at hand. First, we have studied the effect of staying at home when having symptoms for one disease on the final size of the other infection. More specifically, we studied how the following infectious disease parameters influence this effect: basic reproduction numbers, infectious period, fraction of symptomatic cases, number of contacts and the delay between the two epidemic outbreaks. Second, we studied two diseases with different symptom severity, where twice as many people stayed at home when having symptoms of the most severe disease than when having symptoms of the other disease. Both the basic reproduction number and the proportion staying at home were varied.

The paper is organized as follows. In Section 2, we describe the co-infection model configuration, parameter settings, and the scenarios and model variations considered. In Section 3, the results from investigating the effect of behavioral changes due to having symptoms on the model output are presented. Section 4 discusses our main findings and summarizes our conclusions and recommendations for further research.

## 2 Methods

### 2.1 Co-infection model setup

**Fig. 1:**
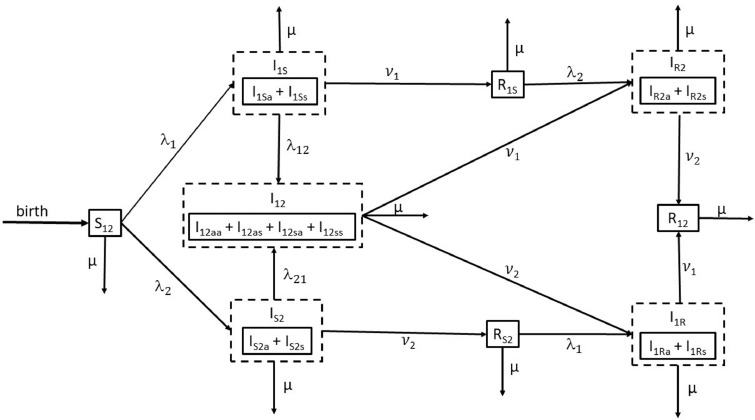
Schematic diagram of the SIR model used in this paper. Model parameters are *μ*: natural mortality rate; *λ*_1_: the marginal force of infection for infection 1; *λ*_2_: the marginal force of infection for infection 2; *λ*_12_: the force of infection for infection 2, conditional on infection 1; *λ*_21_: the force of infection for infection 1, conditional on infection 2; *ν*_1_: the recovery rate for infection 1; *ν*_2_: the recovery rate for infection 2.

The co-infection model used in this paper is an age-structured Susceptible-Infected-Recovered (SIR) compartmental transmission model, describing the joint disease dynamics with regard to two immunizing infections conferring lifelong humoral immunity. The model was implemented in R3.1.1 and R3.3.2 using the deSolve package (Soetaert et al., 2010). In total, the co-infection model consists of 9 different compartments or states, which are described in detail in Table 1. Fig. 1 shows a schematic diagram depicting the different compartments and the flow of individuals between the states in the model.

**Table 1:** Compartments in the SIR model used in this study.

State	Meaning
*S*_12_	Susceptible for both infections
*I*_1*S*_	Infected by pathogen 1, susceptible for infection by pathogen 2
	*I*_1*Sa*_: asymptomatic infection; *I*_1*Ss*_: symptomatic infection
*I*_*S*2_	Infected by pathogen 2, susceptible for infection by pathogen 1
	*I*_*S*2*a*_: asymptomatic infection; *I*_*S*2*s*_: symptomatic infection
*I*_12_	Co-infection
	*I*_12*aa*_: asymptomatic; *I*_12*as*_: only symptoms of disease 2;
	*I*_12*sa*_: only symptoms of disease 1; *I*_12*ss*_: symptoms of both diseases
*I*_1*R*_	Infected by pathogen 1, recovered from infection by pathogen 2
	*I*_1*Ra*_: asymptomatic infection; *I*_1*Rs*_: symptomatic infection
*I*_*R*2_	Infected by pathogen 2, recovered from infection by pathogen 1
	*I*_*R*2*a*_: asymptomatic infection; *I*_*R*2*s*_: symptomatic infection
*R*_1*S*_	Recovered from infection by pathogen 1, susceptible for infection by pathogen 2
*R*_*S*2_	Recovered from infection by pathogen 2, susceptible for infection by pathogen 1
*R*_12_	Recovered from both infections

In particular, these flows can be described using a system of partial differential equations (PDEs), in age (a) and time (t):

$$\begin{array}{ccc} & & \frac{\partial S_{12}\left(a,t\right)}{\partial a}+\frac{\partial S_{12}\left(a,t\right)}{\partial t}=-\left(\lambda_{1}\left(a,t\right)+\lambda_{2}\left(a,t\right)+\mu \left(a\right)\right)S_{12}\left(a,t\right)\\ & & \frac{\partial I_{1S}\left(a,t\right)}{\partial a}+\frac{\partial I_{1S}\left(a,t\right)}{\partial t}=\lambda_{1}\left(a,t\right)S_{12}\left(a,t\right)\\ & & \;\;-\;\left(\mu \left(a\right)+\lambda_{12}\left(a,t\right)+\nu_{1}\right)I_{1S}\left(a,t\right)\\ & & \frac{\partial I_{S2}\left(a,t\right)}{\partial a}+\frac{\partial I_{S2}\left(a,t\right)}{\partial t}=\lambda_{2}\left(a,t\right)S_{12}\left(a,t\right)\\ & & \;\;-\;\left(\mu \left(a\right)+\lambda_{21}\left(a,t\right)+\nu_{2}\right)I_{S2}\left(a,t\right)\\ & & \frac{\partial I_{12}\left(a,t\right)}{\partial a}+\frac{\partial I_{12}\left(a,t\right)}{\partial t}=\lambda_{21}\left(a,t\right)I_{S2}\left(a,t\right)+\lambda_{12}\left(a,t\right)I_{1S}\left(a,t\right)\\ & & \;\;-\;\left(\nu_{1}+\nu_{2}+\mu \left(a\right)\right)I_{12}\left(a,t\right)\\ & & \frac{\partial I_{1R}\left(a,t\right)}{\partial a}+\frac{\partial I_{1R}\left(a,t\right)}{\partial t}=\lambda_{1}\left(a,t\right)R_{S2}\left(a,t\right)+\nu_{2}I_{12}\left(a,t\right)\\ & & \;\;-\;\left(\mu \left(a\right)+\nu_{1}\right)I_{1R}\left(a,t\right)\\ & & \frac{\partial I_{R2}\left(a,t\right)}{\partial a}+\frac{\partial I_{R2}\left(a,t\right)}{\partial t}=\lambda_{2}\left(a,t\right)R_{1S}\left(a,t\right)+\nu_{1}I_{12}\left(a,t\right)\\ & & \;\;-\;\left(\mu \left(a\right)+\nu_{2}\right)I_{R2}\left(a,t\right)\\ & & \frac{\partial R_{1S}\left(a,t\right)}{\partial a}+\frac{\partial R_{1S}\left(a,t\right)}{\partial t}=\nu_{1}I_{1S}\left(a,t\right)-\left(\lambda_{2}\left(a,t\right)+\mu \left(a\right)\right)R_{1S}\left(a,t\right)\\ & & \frac{\partial R_{S2}\left(a,t\right)}{\partial a}+\frac{\partial R_{S2}\left(a,t\right)}{\partial t}=\nu_{2}I_{S2}\left(a,t\right)-\left(\lambda_{1}\left(a,t\right)+\mu \left(a\right)\right)R_{S2}\left(a,t\right)\\ & & \frac{\partial R_{12}\left(a,t\right)}{\partial a}+\frac{\partial R_{12}\left(a,t\right)}{\partial t}=\nu_{2}I_{R2}\left(a,t\right)+\nu_{1}I_{1R}\left(a,t\right)-\mu \left(a\right)R_{12}\left(a,t\right) \end{array}$$

where *λ*_1_(*a, t*), *λ*_2_(*a, t*), *λ*_12_(*a, t*) and *λ*_21_(*a, t*) are the age- and time-dependent marginal and conditional forces of infection (FOI); *μ*(*a*) is the age-dependent natural death rate; *ν*_1_ and *ν*_2_ are the recovery rates which are assumed to be constant.

Put $I_{1}=I_{1S}+I_{12}+I_{1R}$ and $I_{2}=I_{S2}+I_{12}+I_{R2}$. The age- and time-dependent forces of infection are give by Anderson and May (1992):

$$\begin{array}{c} \lambda_{1}\left(a,t\right)=\int_{0}^{\infty }\beta_{1}\left(a,a^{\prime }\right)I_{1}\left(a^{\prime },t\right)da^{\prime }\\ \lambda_{2}\left(a,t\right)=\int_{0}^{\infty }\beta_{2}\left(a,a^{\prime }\right)I_{2}\left(a^{\prime },t\right)da^{\prime }\\ \lambda_{12}\left(a,t\right)=\int_{0}^{\infty }\beta_{12}\left(a,a^{\prime }\right)I_{2}\left(a^{\prime },t\right)da^{\prime }\\ \lambda_{21}\left(a,t\right)=\int_{0}^{\infty }\beta_{21}\left(a,a^{\prime }\right)I_{1}\left(a^{\prime },t\right)da^{\prime } \end{array}$$

where *β*_1_(*a, a*′), *β*_2_(*a, a*′), *β*_12_(*a, a*′), *β*_21_(*a, a*′) are the transmission rates, i.e. the average per capita rates at which a susceptible individual of age a makes effective contact with an infected individual of age a’, per unit time; *I*_1_(*a*′, *t*) and *I*_2_(*a*′, *t*) denote the total number of infected individuals of age a’ at time t. If the population is divided into K age categories, the age- and time-dependent forces of infection are given by (discretized version of the equations above) Hens et al. (2012)

$$\begin{array}{c} \lambda_{1}\left(k,t\right)=\sum_{k^{\prime }=0}^{K}\beta_{1}\left(k,k^{\prime }\right)I_{1}\left(k^{\prime },t\right)\\ \lambda_{2}\left(k,t\right)=\sum_{k^{\prime }=0}^{K}\beta_{2}\left(k,k^{\prime }\right)I_{2}\left(k^{\prime },t\right)\\ \lambda_{12}\left(k,t\right)=\sum_{k^{\prime }=0}^{K}\beta_{12}\left(k,k^{\prime }\right)I_{2}\left(k^{\prime },t\right)\\ \lambda_{21}\left(k,t\right)=\sum_{k^{\prime }=0}^{K}\beta_{21}\left(k,k^{\prime }\right)I_{1}\left(k^{\prime },t\right) \end{array}$$

where *β*_1_(*k, k*′), *β*_2_(*k, k*′), *β*_12_(*k, k*′), *β*_21_(*k, k*′) are the transmission rates, i.e. the average per capita rates at which a susceptible individual in age category k makes effective contact with an infected individual in age category k’, per unit time; *I*_1_(*k*′, *t*) and *I*_2_(*k*′, *t*) denote the total number of infected individuals in age category k’ at time t.

### 2.2 Parameter configuration

#### 2.2.1 Population and age structure

In this study, simulations were run for the Belgian population aged 0–85 years in 2012 (population size = 10,785,904, of which 22% are school-aged children (0–18 years) and 78% are adults (19–85 years)) FOD (Federale overheidsdienst economie afdeling statistiek (2017). Because of model simplicity, we assume type I mortality and a life expectancy of 85 years. This means that everyone survives up to the age of 85 years and then immediately dies. Therefore, the maximum age in the model is 85 years. Type I mortality is a reasonable approximation of the mortality function for high-income countries (Anderson and May, 1992).

In the simulations, individuals in the population with ages ranging from 0 to 85 years in the different compartments are divided into one year age categories. Model parameters are assumed to be age-specific and are allowed to differ by calendar time (see also next paragraph). The main results are illustrated for two wide age classes: school-aged children (0–18 years) and adults (19–85 years). The division into these two age categories is driven by the important, but different role these two age classes have in the spread of influenza. While children mainly spread the epidemic by their high contact rates at school, adults mainly do this by commuting and traveling (Apolloni, Poletto, Colizza, 2013, De Luca, Van Kerckhove, Coletti, Poletto, Bossuyt, Hens, Colizza, 2018).

#### 2.2.2 Social contact matrices

The age- and time-specific marginal and conditional forces of infection (FOI) *λ*_1_(*a, t*), *λ*_2_(*a, t*), *λ*_12_(*a, t*), and *λ*_21_(*a, t*), were related to the social contact data using the mass action approach by Wallinga and colleagues (2006). The corresponding values of the FOI were calculated for various hypothesized values of the basic reproduction number for each of the two infections, that is, the average number of secondary infections produced by a single ‘typical’ infectious individual during his/her entire infectious period when introduced in a fully susceptible population. When describing a co-infection model, the system is driven by two basic reproduction numbers *R*_0,1_ and *R*_0,2_. The basic reproduction numbers do not depend on the number of co-infections, since co-infections are rare at the start of an epidemic. In this study, four different mixing matrices were constructed based on social contact survey data:

• *C_aa_*: asymptomatic mixing matrix describing the age-specific mixing behavior of asymptomatic individuals;
• *C_sa_* and *C_as_*: mixing matrix for individuals only having symptoms of disease 1 resp. disease 2;
• *C_ss_*: mixing matrix when having symptoms of both diseases under study.

Data from the social contact survey studied in Van Kerckhove et al. (2013) were used to construct 2 × 2 contact matrices *C^A^, C^S^* and $C_{h}^{S}$ for the two age categories defined previously. *C^A^* is the asymptomatic contact matrix, which is assumed to be the same as the contact matrix for ‘healthy’ individuals (i.e., ‘healthy’ with regard to the infections at hand). Furthermore, *C^S^* is the contact matrix for symptomatic individuals not staying at home and $C_{h}^{S}$ the contact matrix for symptomatic individuals staying at home. The contact matrices, describing the daily age-specific contact rates, are given by:

$$C^{A}=\left(\begin{array}{cc} 7.418e-07 & 1.070e-07\\ 8.839e-08 & 1.609e-07 \end{array}\right),$$

$$C^{S}=\left(\begin{array}{cc} 1.203e-07 & 6.830e-08\\ 4.438e-08 & 5.636e-08 \end{array}\right),$$

$$C_{h}^{S}=\left(\begin{array}{cc} 8.698e-08 & 5.650e-08\\ 4.012e-08 & 3.677e-08 \end{array}\right).$$

If we compare the contact matrices, we can derive that having symptoms decreases the contact rates with 36%-84%. Staying at home decreases the symptomatic contact rates with 10%–34%.

Let *p*_1_ and *p*_2_ represent the proportions of individuals staying at home when having symptoms of disease 1 and 2, respectively, and let *p*_12_ be the proportion of individuals staying at home when having symptoms of both diseases. In the first scenario, where people only stay at home for the most severe disease, we assume that $p_{12}=p_{1}$. In the second scenario, where people stay at home for both diseases, we assume that *p*_12_ will be larger than *p*_1_ and *p*_2_. In this study, we define *p*_12_ as $p_{1}+p_{2}-p_{1}p_{2},$ so that *p*_12_ is always the largest of the three proportions *p*_1_, *p*_2_ and *p*_12_. According to the aforementioned notation, the social contact matrices are given by: $C_{aa}=C^{A}$; $C_{sa}=p_{1}C_{h}^{S}+\left(1-p_{1}\right)C^{S}$; $C_{as}=p_{2}C_{h}^{S}+\left(1-p_{2}\right)C^{S}$; and $C_{ss}=p_{12}C_{h}^{S}+\left(1-p_{12}\right)C^{S}$.

When *ϕ*_1_ (resp. *ϕ*_2_) is the proportion of symptomatic cases of infection 1 (resp. infection 2), then the age-specific transmission rates can be calculated from the mixing matrices as follows (Wallinga et al., 2006)

$$\begin{array}{cc} \beta_{1}\left(k,k^{\prime }\right)= & \left(1-\phi_{1}\right)q_{1a}C_{aa}\left(k,k^{\prime }\right)+\phi_{1}q_{1s}C_{sa}\left(k,k^{\prime }\right)\\ \beta_{2}\left(k,k^{\prime }\right)= & \left(1-\phi_{2}\right)q_{2a}C_{aa}\left(k,k^{\prime }\right)+\phi_{2}q_{2s}C_{as}\left(k,k^{\prime }\right)\\ \beta_{12}\left(k,k^{\prime }\right)= & \left(1-\phi_{1}\right)\left(1-\phi_{2}\right)q_{2a}C_{aa}\left(k,k^{\prime }\right)+\left(1-\phi_{1}\right)\phi_{2}q_{2s}C_{as}\left(k,k^{\prime }\right)\\ & +\phi_{1}\left(1-\phi_{2}\right)q_{2a}C_{sa}\left(k,k^{\prime }\right)+\phi_{1}\phi_{2}q_{2s}C_{ss}\left(k,k^{\prime }\right)\\ \beta_{21}\left(k,k^{\prime }\right)= & \left(1-\phi_{1}\right)\left(1-\phi_{2}\right)q_{1a}C_{aa}\left(k,k^{\prime }\right)+\phi_{1}\left(1-\phi_{2}\right)q_{1s}C_{sa}\left(k,k^{\prime }\right)\\ & +\left(1-\phi_{1}\right)\phi_{2}q_{1a}C_{as}\left(k,k^{\prime }\right)+\phi_{1}\phi_{2}q_{1s}C_{ss}\left(k,k^{\prime }\right) \end{array}$$

where *q*_1*a*_, *q*_1*s*_, *q*_2*a*_ and *q*_2*s*_ are disease-specific proportionality factors for asymptomatic infection 1, symptomatic infection 1, asymptomatic infection 2 and symptomatic infection 2.

#### 2.2.3 Model variations and scenarios

In a first baseline scenario, simulations were run for two infections starting at the same time which both have an infectious period of 7 days. For both infections 60% of infections were symptomatic and symptomatic cases were supposed to be three times as infectious as asymptomatic cases. The basic reproduction numbers *R*_0,1_ and *R*_0,2_ were equal for both diseases and $R_{0}=R_{0,1}=R_{0,2}$ was varied between 1.5 and 6.5, with steps of size 1. The percentage of individuals staying at home when having symptoms of disease 1 was varied between 0% and 100%, with steps of size 5%. People were supposed not to stay at home when having symptoms of the second disease. The following model variations were applied to this scenario:

• infectious period of both infections respectively 14 days and 21 days;
• infectious period of one of the infections respectively 14 days and 21 days (and the other 7 days);
• for both infections symptomatic cases are six times (respectively nine times) as infectious as asymptomatic cases;
• for one infection symptomatic cases are six times (respectively nine times) as infectious as asymptomatic cases (and for the other three times as infectious);
• for both infections 90% (respectively 30%) of the infected individuals are symptomatic;
• for one infection 90% (respectively 30%) of the infected individuals are symptomatic (and for the other 60% are symptomatic);
• asymptomatic individuals would have the same mixing patterns as symptomatic individuals not staying at home ($C^{A}=C^{S},$ both matrices are equal to the symptomatic contact matrix);
• a difference of 0.3 between the basic reproduction numbers of the two diseases;
• a delay of one month between the two diseases.

As a second scenario, two infections were studied, for which the proportion staying at home when having symptoms of the less severe disease was half of the proportion staying at home when having symptoms for the other disease. For all scenarios in which both infections are introduced simultaneously, the model was initialized with 1 co-infected person of 10 years old and the remainder of the population was considered susceptible for both infections. For the scenarios with a delay between the starting times of the two infections, the start of infection 1 (resp. infection 2) was initialized with 1 person of 10 years old, mono-infected by pathogen 1 (resp. pathogen 2) and still susceptible for the other infection.

### 2.3 Solving the system of PDEs – Method of lines

In order to numerically solve the system of PDEs for the co-infection model presented in Fig. 1, we rely on the method of lines (Schiesser, 2012) in which the age dimension is discretized and only the time dimension remains continuous. Consequently, the method of lines leads to a system of ODEs that can be solved by means of a numerical method for initial value ODEs. For more details regarding the method of lines, we refer to Schiesser (2012).

## 3 Results

In this section, we discuss the results of our simulation approach. First, we investigated the effect of staying at home when having symptoms of one disease on the dynamics of the other infection. Second, the influence of the following model parameters on the observed effect was studied: the basic reproduction number, the infectious period, the infectiousness of symptomatic versus asymptomatic individuals, the proportion of cases being symptomatic, the number of contacts and delays between the start of the two infections. Third, the effect of staying at home for two diseases where twice as many people stay at home when having symptoms of the most severe disease compared to the other disease was investigated.

The observed results are explained by comparing the dynamic profiles of the infections, including the peak time of infection.

### 3.1 Influence of home isolation when having symptoms of one disease

**Fig. 2:**
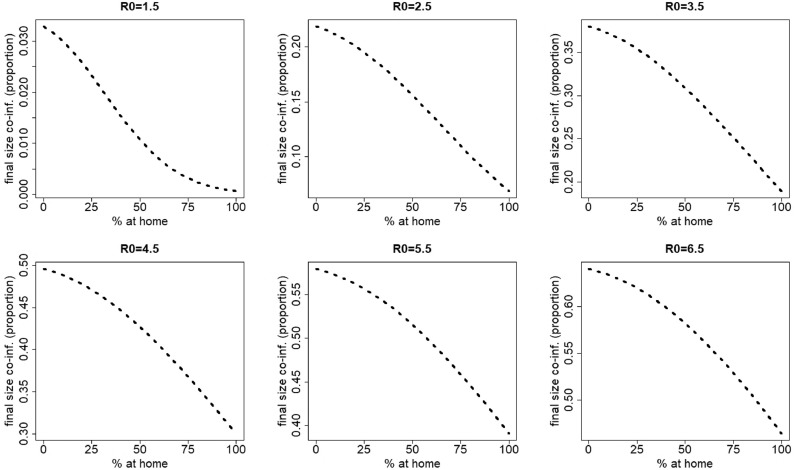
Final size of co-infection (as a proportion of the total population) against the percentage staying at home when having symptoms of disease 1 for different values of $R_{0}=R_{0,1}=R_{0,2}$. The parameters used are those of the first baseline scenario.

Changes in contact behavior by staying at home when having symptoms of the most severe disease (disease 1) induces the final size of co-infection to decrease (Fig. 2).

**Fig. 3:**
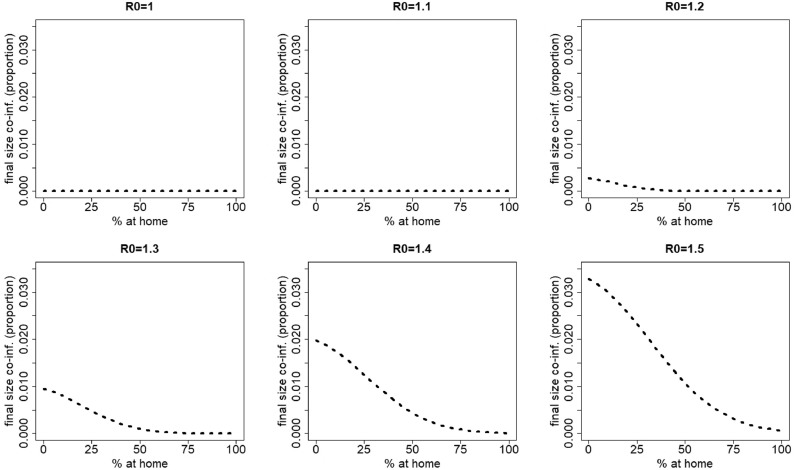
Final size of co-infection (as a proportion of the total population) against the percentage staying at home when having symptoms of disease 1 for $R_{0}=R_{0,1}=R_{0,2}$ varying between 1 and 1.5. In contrast to Fig. 2, the same scale is used on the vertical axis. The parameters used are those of the first baseline scenario.

Fig. 3 graphically depicts the effect of *R*_0_ on the total number of co-infections for a range of *R*_0_ values between 1 and 1.5. Here, we observe that staying at home counteracts the natural increase of the final size of co-infections with increasing *R*_0_.

**Fig. 4:**
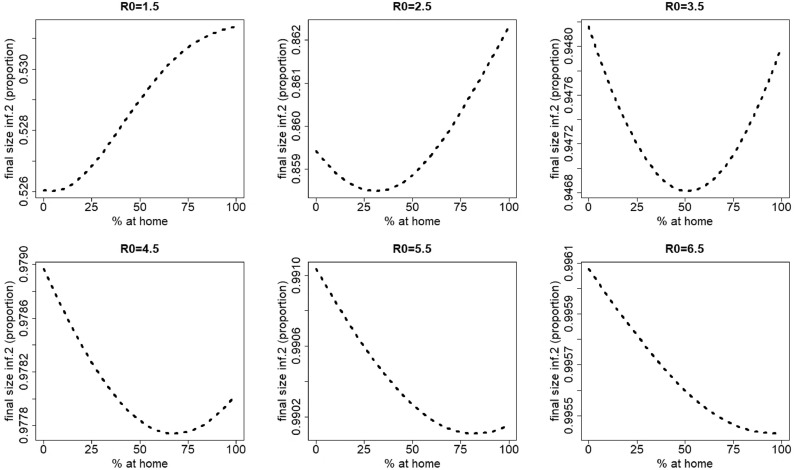
Final size of infection 2 (as a proportion of the total population) against the percentage staying at home when having symptoms of disease 1 for different values of $R_{0}=R_{0,1}=R_{0,2}$. The parameters used are those of the first baseline scenario.

**Fig. 5:**
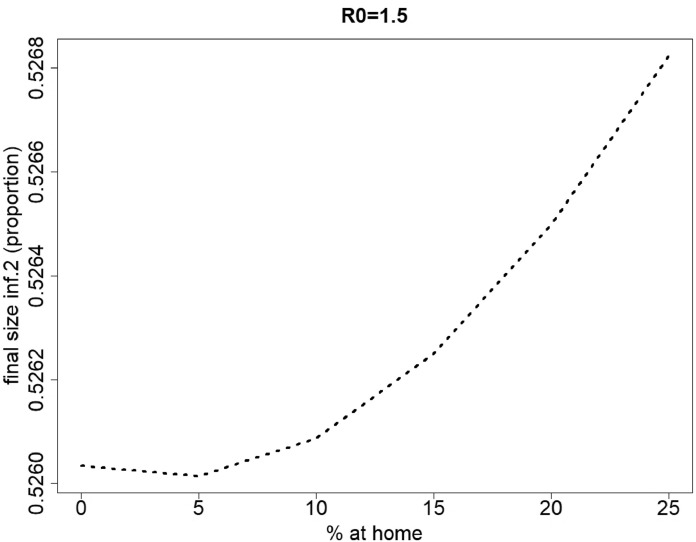
Final size of infection 2 (as a proportion of the total population) against the percentage staying at home when having symptoms of disease 1 for $R_{0}=R_{0,1}=R_{0,2}=1.5$ and percentages staying at home ranging from 0% up to 25% in steps of size 5%. The parameters used are those of the first baseline scenario.

For infection 2, different scenarios can be observed, depending on the value of the basic reproduction number. Fig. 4 depicts the final size of infection 2 for varying percentages of individuals staying at home when having symptoms of disease 1 (*p*_1_ ranges from 0% up to 100% in steps of size 5%), and varying values of the basic reproduction number (*R*_0_ ranges from 1.5 up to 6.5 in steps of size one). For small to moderate values of *R*_0_ ($R_{0}=1.5-5.5$) the final size of infection 2 initially decreases with an increasing percentage (*p*_1_) of people staying at home for disease 1 (for $R_{0}=1.5,$ see also Fig. 5). After reaching a minimum, the final size of infection 2 increases with increasing *p*_1_. The value of *p*_1_ corresponding with the minimal final size of infection 2 increases with increasing *R*_0_. However, for high *R*_0_ values, hence, more contagious pathogens, the final size of infection 2 decreases with increasing *p*_1_ values.

**Fig. 6:**
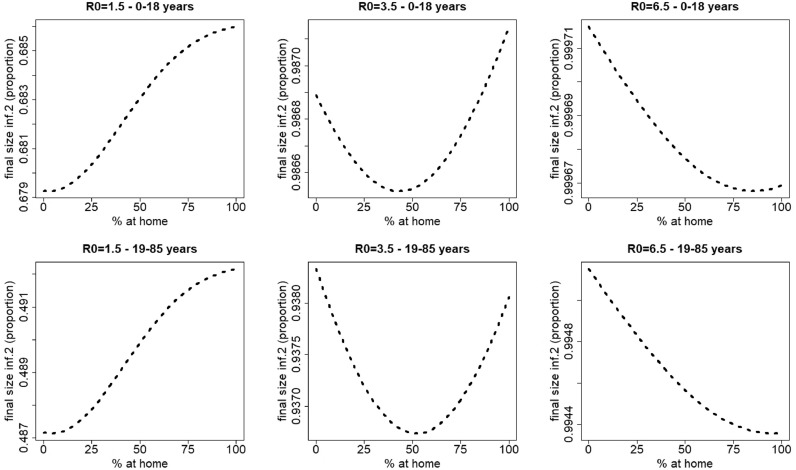
Final size of infection 2 against the percentage staying at home when having symptoms of disease 1 for different values of $R_{0}=R_{0,1}=R_{0,2}$. Left: $R_{0}=1.5,$ middle: $R_{0}=3.5,$ right: $R_{0}=6.5$. Upper panel: school-aged children (0–18 years), as a proportion of the total number of children, lower panel: adults (19–85 years), as a proportion of the total number of adults. The parameters used are those of the first baseline scenario.

Fig. 6 shows the results of Fig. 4, stratified in the age classes 0–18 years (school-aged children) and 19–85 years (adults) for $R_{0}=$ 1.5, 3.5 and 6.5. The qualitative effects of home isolation are similar for both age classes.

**Fig. 7:**
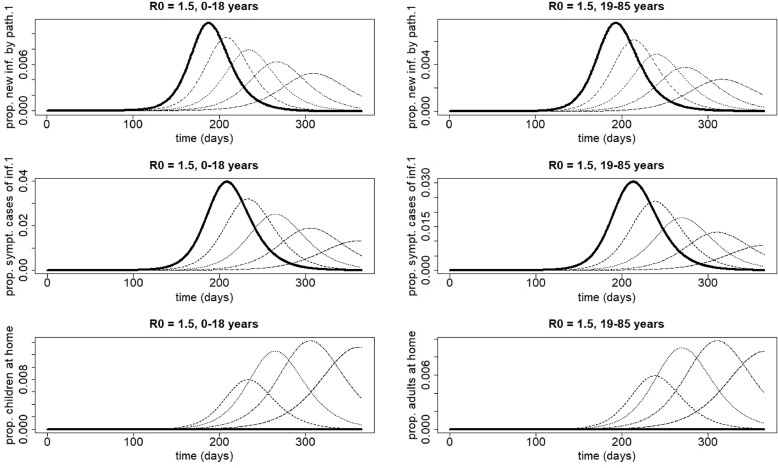
Influence of home isolation for disease 1 only, for school-aged children (age class 0−18 years, left figures) and adults (age class 19−85 years, right figures) in case of two infections with an infectious period of 7 days and $R_{0}=R_{0,1}=R_{0,2}=1.5$. The parameters used are those of the first baseline scenario. Solid line: 0% at home, dashed line: 25% at home, dotted line: 50% at home, dotdashed line: 75% at home, longdashed line: 100% at home. Upper panel, left: proportion of newly infected school-aged children with pathogen 1. Because there is no home isolation for disease 2, the number of new infections with pathogen 2 coincides with the solid line; middle panel, left: proportion of symptomatic cases of infection 1 in school-aged children ; lower panel, left: proportion of school-aged children staying at home. Upper panel, right: proportion of new infections with pathogen 1 in adults. Because there is no home isolation for disease 2, the proportion of new infections with pathogen 2 coincides with the solid line; middle panel, right: proportion of symptomatic cases of infection 1 in adults; lower panel, right: proportion of symptomatic cases of infection 1 staying at home in adults.

From Fig. 7 (upper panel), it can be observed that staying at home when having symptoms of the most severe infection (infection 1) leads to a shift of the peak time of new infections with pathogen 1. When we define *R*_*h*,1_ as the value of *R*_0,1_ after the introduction of staying-at-home behavior, this shift can be explained by *R*_*h*,1_ < *R*_0,1_ (e.g. for $R_{0,1}=1.5,$ the value of *R*_*h*,1_ in the basis scenario is 1.44, 1.39, 1.33 and 1.28 for 25%, 50%, 75% and 100% of individuals with symptoms of disease 1 and staying at home respectively). The shift of the peak time increases with increasing *p*_1_ values. As a consequence, at the peak time of the less severe disease (infection 2), which equals the peak time of infection 1 in case of no home isolation (solid line Fig. 7, upper panel), the number of symptomatic cases of infection 1 decreases with increasing *p*_1_ (middle panel). Furthermore, the number of people staying at home at the peak time of infection 2 decreases with increasing *p*_1_ (lower panel). This means that, as *p*_1_ increases, the number of symptomatic individuals with infection 1 (who, whether they stay at home or not, have fewer contacts than healthy or asymptomatic individuals) around the peak of infection 2 decreases. As a consequence, people will have on average more contacts and will have a higher probability to acquire infection 2. This explains the increasing trend in the final size of infection 2.

When comparing the left with the right figures in Fig. 7, it can be observed that similar scenarios occur for both age classes.

**Fig. 8:**
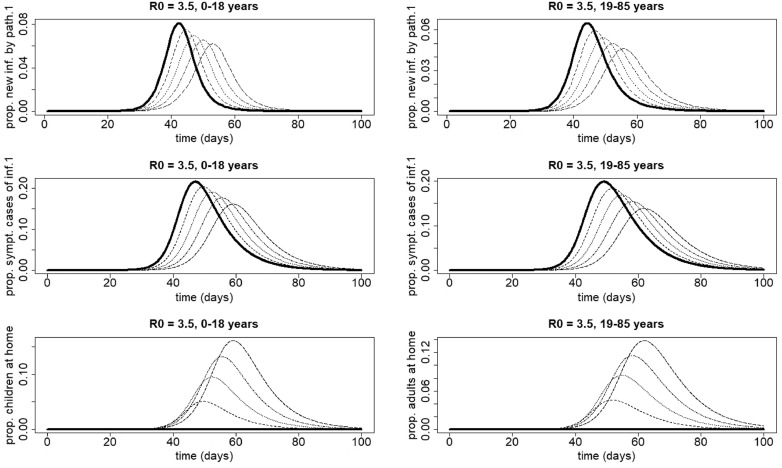
Influence of home isolation for disease 1 only, for school-aged children (age class 0–18 years, left figures) and adults (age class 19–85 years, right figures) in case of two infections with an infectious period of 7 days and $R_{0}=R_{0,1}=R_{0,2}=3.5$. The parameters used are those of the first baseline scenario. Solid line: 0% at home, dashed line: 25% at home, dotted line: 50% at home, dotdashed line: 75% at home, longdashed line: 100% at home. Upper panel, left: proportion of newly infected school-aged children with pathogen 1. Because there is no home isolation for disease 2, the number of new infections with pathogen 2 coincides with the solid line; middle panel, left: proportion of symptomatic cases of infection 1 in school-aged children; lower panel, left: proportion of school-aged children staying at home. Upper panel, right: proportion of new infections with pathogen 1 in adults. Because there is no home isolation for disease 2, the proportion of new infections with pathogen 2 coincides with the solid line; middle panel, right: proportion of symptomatic cases of infection 1 in adults; lower panel, right: proportion of symptomatic cases of infection 1 staying at home in adults.

For larger values of *R*_0_ (e.g. $R_{0}=3.5$), the shift of the peak time of new infections with pathogen 1 becomes smaller (compare Fig. 7 with Fig. 8). At the peak time of infection 2, the decrease of the number of people staying at home occurs over a smaller interval (compare Fig. 7 with Fig. 8). As a consequence, the increasing part of the graph of the final size of infection 2 in Fig. 4 becomes smaller with increasing *R*_0_ values.

### 3.2 Influence of model parameters on the observed effects

The following model parameters have little or no influence on the qualitative effects observed in Section 3.1 when varied together for both infections : the infectious period, the infectiousness of symptomatic versus asymptomatic cases, the fraction of symptomatic cases (see Supplementary Material, Figs. S1-S3). Furthermore staying at home for disease 1 has limited or no effect on disease 2 when the diseases have infectious periods that differ one or more weeks (see Figs. S6-S7). Varying the infectiousness of symptomatic versus asymptomatic cases for only one disease, or varying the percentage of symptomatic cases for disease 2 has limited effect on the qualitative effects observed in Section 3.1 (see Figs. S8, S9 and S11). The effects observed in Section 3.1 increase with an increasing percentage of symptomatic cases for disease 1 (see Fig. S10).

**Fig. 9:**
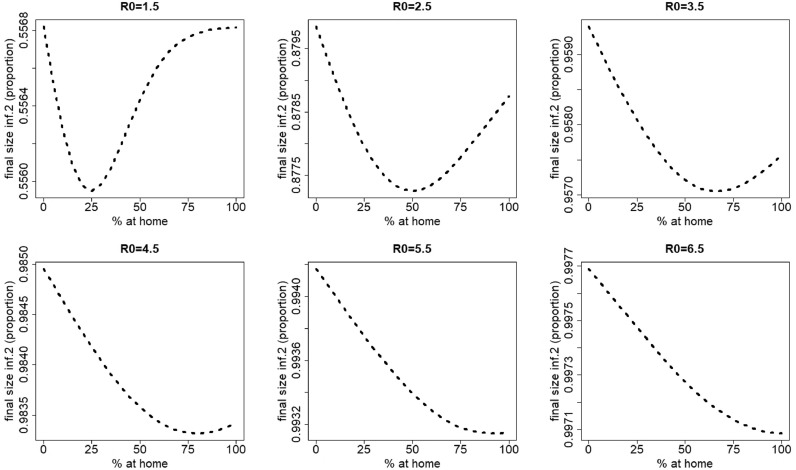
Influence of contact matrices on the behavior observed in Section 3.1. Final size of infection 2 (as a proportion of the total population) against the percentage staying at home when having symptoms of disease 1 for different values of $R_{0}=R_{0,1}=R_{0,2}$ when asymptomatic individuals would have the same mixing patterns as symptomatic individuals not staying at home ($C^{A}=C^{S},$ both matrices are equal to the symptomatic contact matrix). The parameters used are those from the first baseline scenario.

If asymptomatic cases had the same mixing patterns as symptomatic cases of infection 1 not staying at home, the final size of infection 2 would never be higher when staying at home than without home isolation. The interval that shows an increasing final size of infection 2 with increased home isolation becomes smaller (compare Figs. 4 and 9, especially for $R_{0}=$ 1.5 and 2.5). This smaller effect of staying at home, compared to the basis scenario, can be explained as follows. When $C^{A}=C^{S},$ we still have *R*_*h*,1_ < *R*_0,1_ and the peak shift of infection 1. However, while in the basis scenario there were two groups having fewer contacts than healthy individuals (symptomatic cases of disease 1 staying at home and symptomatic cases not staying at home), in this scenario we have only one group with fewer contacts than healthy individuals (symptomatic cases staying at home). So the average number of contacts will also increase around the peak of infection 2 because of the shift in peak of infection 1, but to a lower extent.

**Fig. 10:**
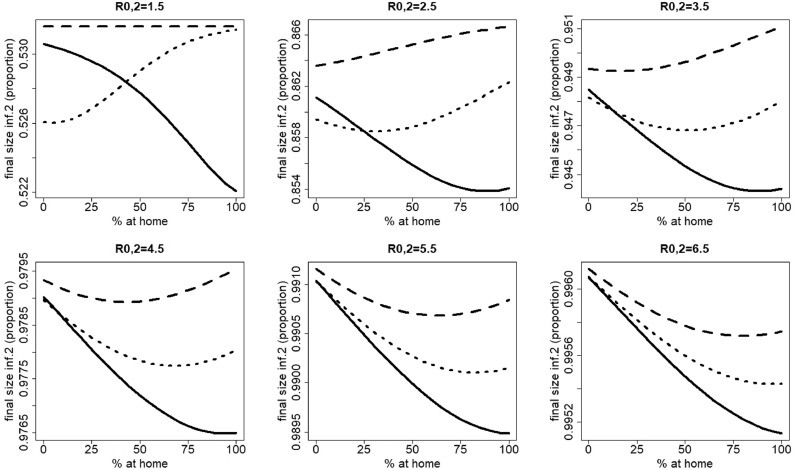
Influence of the difference in *R*_0_ between pathogen 1 and pathogen 2 on the behavior observed in Section 3.1. Dotted line: $R_{0,1}=R_{0,2}$; dashed line: $R_{0,1}=R_{0,2}-0.3$; solid line: $R_{0,1}=R_{0,2}+0.3$. All other parameters were taken from the first baseline scenario.

When *R*_0_ of pathogen 1 is smaller (resp. larger) than *R*_0_ of pathogen 2, a decrease of the final size of infection 2 with *p*_1_ starts to occur at higher (resp. lower) values of *R*_0_ for pathogen 2, compared to two pathogens with equal *R*_0_ (compare Figs. 4 and 10).

**Fig. 11:**
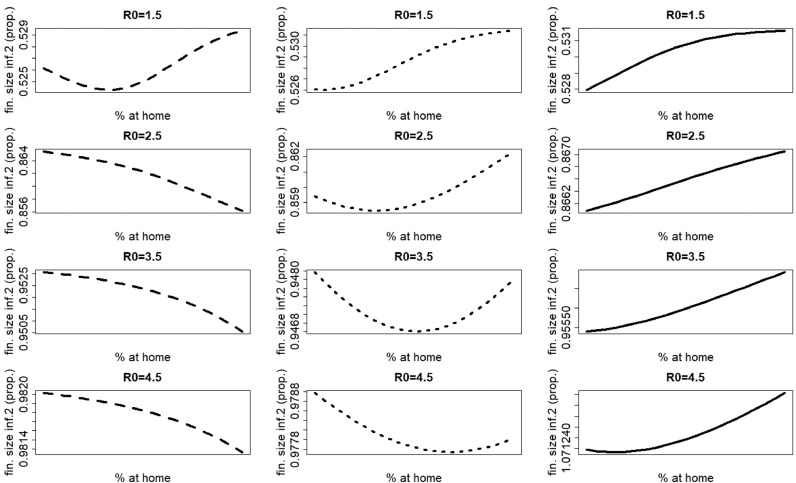
Influence of the delay between the start of the two infections on the behavior observed in Section 3.1. Dashed line: infection 1 starts one month earlier than infection 2; dotted line: infections start at the same time; solid line: infection 1 starts one month later than infection 2. All other parameters were taken from the first baseline scenario. $R_{0}=R_{0,1}=R_{0,2}$.

In case infection 1 starts earlier (resp. later) than infection 2, a decrease of the final size of infection 2 with *p*_1_ starts to occur at smaller (resp. larger) *R*_0_ (Fig. 11), compared to two infections with equal starting time.

### 3.3 Influence of symptom severity

A more realistic setting, in which twice as many people stay at home when having symptoms of the most severe disease (disease 1) than when having symptoms of the less severe disease (disease 2)($p_{1}=2\cdot p_{2}$), was simulated. More specifically, the following scenario was studied in detail:

• Based on the 2009 A/H1N1pdm influenza epidemic, we assume that he percentage staying at home when having symptoms of the most severe disease (*p*_1_) is 70% (Kim Van Kerckhove, personal communication).
• The percentage staying at home when having symptoms of the less severe disease (*p*_2_) is 35%.
• Symptomatic individuals are three times as infectious as asymptomatic individuals (Van Kerckhove et al., 2013).
• For both diseases, 66% of the infections are symptomatic (Van Kerckhove et al., 2013).
• Both diseases have $R_{0}=1.5,$ an infectious period of 7 days and start at the same time.

**Fig. 12:**
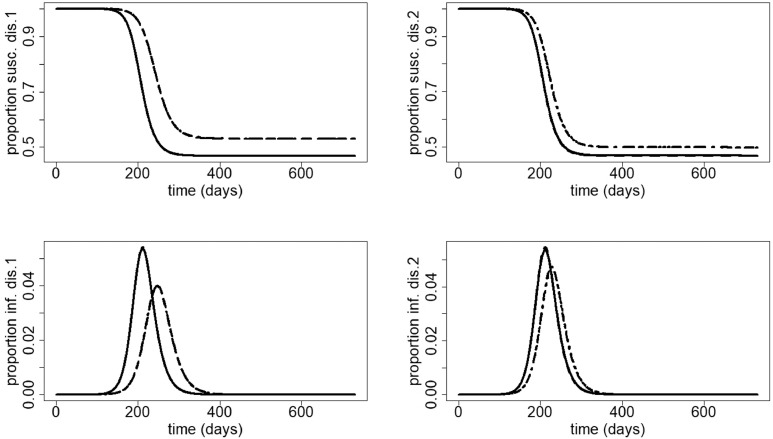
Second scenario. First row: proportion of susceptible for disease 1 (left) and disease 2 (right); second row: proportion of infected with disease 1 (left) and disease 2 (right). Scenarios: no home isolation (solid); 70% symptomatic cases at home for disease 1, no home isolation for disease 2 (dashed); 35% symptomatic cases at home for disease 2, no home isolation for disease 1 (dotted); 70% symptomatic cases at home for disease 1, 35% symptomatic cases at home for disease 2 (dotdashed). The parameters used are those described in Section 3.3. In the left figures, the following lines coincide: solid and dotted; dashed and dotdashed, suggesting that staying at home for disease 2 has limited effect on the dynamics of infection 1. In the right figures, the following lines coincide: solid and dashed; dotted and dotdashed, suggesting that staying at home for disease 1 has limited effect on the dynamics of infection 2.

Fig. 12 (left figures) shows that for infection 1, the numbers of susceptible and infected are almost equal when not staying at home compared to the situation in which 35% of symptomatic individuals having disease 2 stay at home (and there is no home isolation for disease 1). This suggests that the impact of staying at home for disease 2 on the dynamics of infection 1 is negligible. A higher number of susceptible and a lower number of infected individuals are observed for infection 1 when 70% of symptomatic individuals with disease 1 stay at home (and there is no home isolation for disease 2). This means that staying at home for disease 1 has a large positive effect on the spread of epidemic 1. Furthermore, the peak time of infection shifts to the right (the peak of epidemic 1 is delayed). A similar scenario is observed when 70% of symptomatic individuals with disease 1 and 35% of symptomatic individuals with disease 2 stay at home, again suggesting that home isolation for disease 2 has limited effect on the dynamics of infection 1. For disease 2, Fig. 12 (right figures) shows that the proportion of susceptible (resp. infected) is a bit lower (resp. higher) when 70% symptomatic individuals with disease 1 stay at home (and there is no home isolation for disease 2) than when there is no home isolation for both diseases. This means that staying at home for disease 1 has a very small but negative effect on the spread of epidemic 2. A significantly higher (resp. lower) proportion of susceptible (resp. infected) for infection 2 is observed when 35% of symptomatic individuals with disease 2 stay at home (and there is no home isolation for disease 1). This suggests that staying at home for disease 2 has a large positive effect on the spread of epidemic 2. Furthermore, the peak time of infection shifts to the right (the peak of epidemic 2 is delayed). When 70% of symptomatic individuals with disease 1 and 35% of symptomatic individuals with disease 2 stay at home, the number of susceptible (resp. infected) is a bit lower (resp. higher) than when only 35% of symptomatic individuals with disease 2 stay at home (and there is no home isolation for disease 1). This again suggests a limited negative effect of home isolation for disease 1 on the spread of epidemic 2.

**Fig. 13:**
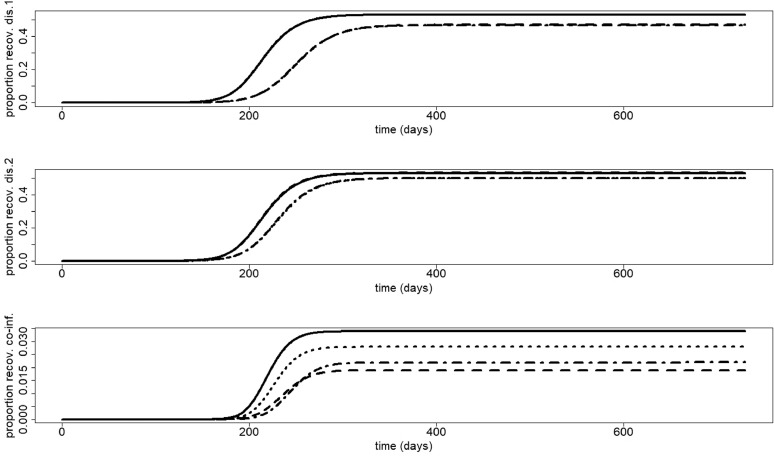
Second scenario. Proportion of people recovered from disease 1 (upper panel), disease 2 (middle panel) and co-infection (lower panel). Scenarios: no home isolation (solid); 70% symptomatic cases at home for disease 1, no home isolation for disease 2 (dashed); 35% symptomatic cases at home for disease 2, no home isolation for disease 1 (dotted); 70% symptomatic cases at home for disease 1, 35% symptomatic cases at home for disease 2 (dotdashed). The parameters used are those described in Section 3.3. In the upper panel, the following lines coincide: solid and dotted; dashed and dotdashed. In the middle panel, the following lines coincide: solid and dashed; dotted and dotdashed.

Fig. 13 shows the proportion of individuals recovered from infection 1, infection 2 and co-infections. Like mentioned before, staying at home when having symptoms of only one disease has a significant positive effect on that disease, and a slightly negative effect on the other. When considering co-infections, the most advantageous scenario is staying at home when having symptoms of the most severe disease, followed by staying at home when having symptoms of one of the two diseases.

**Fig. 14:**
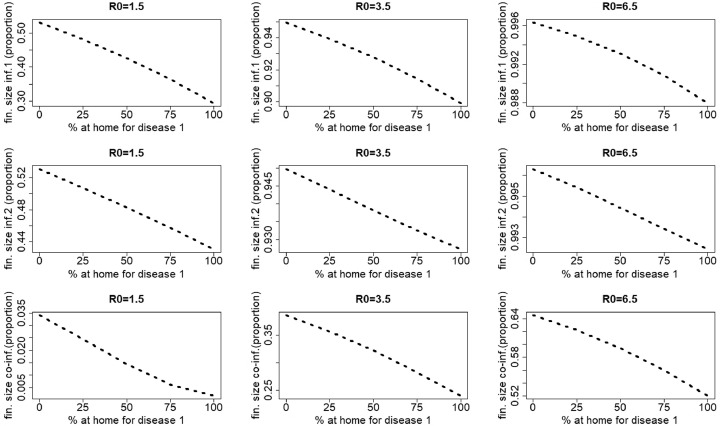
Final size of infection 1 (first row), infection 2 (second row) and co-infections (third row) (as a proportion of the total population) against the percentage staying at home when having symptoms of disease 1 for different values of $R_{0}=R_{0,1}=R_{0,2}$. Left: $R_{0}=1.5,$ middle: $R_{0}=3.5,$ right: $R_{0}=6.5$. The parameters used are those described in Section 3.3.

Fig. 14 shows that in case twice as many people stay at home when having symptoms of the most severe disease than when having symptoms of the other, increasing *p*_1_ and *p*_2_ always decreases the final size of disease 1, disease 2 and co-infections, irrespective of the value of $R_{0}=R_{0,1}=R_{0,2}$.

## 4 Discussion

In this paper, we explored various scenarios of altering behavior, upon contraction of an infection, using a co-infection model. More specifically, we studied the effect of changing social contact behavior on the dynamics and final size of emerging infections, aiming at an improved understanding of social interventions distancing. When studying an influenza-like disease in isolation, Santermans et al. (2017) showed that staying at home leads to a significant reduction of the final size of the disease. However, multiple infectious diseases often circulate within the same period, or with a delay of only a few months between the peak times of the infections. Examples are influenza A and parainfluenza which have coinciding peaks, and RSV and Mycoplasma pneumoniae with a delay of about four months between the peaks (Bollaerts et al., 2013).

Here, we explored two infectious diseases circulating during the same period, where the symptoms of only one of the diseases are severe enough to stay at home. The effect of staying at home for the disease with the severe symptoms on the final size of the other infection was studied. For two diseases with a similar basic reproduction number and a similar infectious period, staying at home for the disease with the severe symptoms can cause a small increase in the final size of the other infection in case of low basic reproduction numbers. This could be explained by a shift in the peak time of infection of the disease with the severe symptoms, resulting in a smaller number of people with less contacts at the peak time of the other infection. This effect was influenced by the mixing patterns, the timing of the two infections and the difference in basic reproduction number between the two pathogens.

The same effects also occur when studying a model with 86 × 86 contact matrices instead of 2 × 2 matrices (see Supplementary Material, Fig. S4), which shows that the observed effects are not an artifact of the model. Moreover, when using 1 × 1 contact matrices, qualitatively similar effects are observed (see Supplementary Material, Fig. S5), suggesting that the effects observed in Section 3.1 do not depend on age-specific heterogeneity in contact behavior. As a consequence, the division into two age groups does not influence the conclusions of this study. Needless to say, this implies that we could simplify the current analysis. However, we believe it is important to consider two age-classes, given that age-heterogeneity has been proven to be relevant when modeling influenza (see e.g. Eames et al., 2012).

The outcome observed in this study relies on the assumptions that asymptomatic individuals have more contacts than symptomatic individuals, and symptomatic individuals have less contacts when staying at home than when not staying at home. These assumptions are realistic and have been confirmed by social contact surveys (Van Kerckhove, Hens, Edmunds, Eames, 2013, Mossong, Hens, Jit, Beutels, Auranen, Mikolajczyk, Massari, Salmaso, Tomba, Wallinga, et al., 2008).

Let *R*_0,1_ and *R*_0,2_ represent the basic reproduction numbers of disease 1 and disease 2 respectively. A larger effect on the change of the final size of infection 2 with increasing *p*_1_ is observed when comparing $R_{0,1}=R_{0,2}$ with $R_{0,1}=R_{0,2}+0.3$ than when comparing $R_{0,1}=R_{0,2}$ with $R_{0,1}=R_{0,2}-0.3$. This is because the two scenarios are not symmetric. Increasing *R*_0,1_ by 0.3 and keeping *R*_0,2_ at its value of Fig. 4 causes a larger shift of the peak time of infection 1 than decreasing *R*_0,1_ by 0.3.

Second, we studied two infectious diseases for which the most severe one induces twice as many symptomatic individuals staying at home than for the other disease. Here, it was observed that no matter what the basic reproductive number is, increasing the proportion staying at home always reduces the final size of both infections, and in particular considerably reduces the number of co-infections.

Our approach has several limitations. First, variation of immunity, which can have a considerable impact on the attack rates and epidemic peaks (Woolthuis et al., 2017), was not taken into account. Second, the study was restricted to a limited number of model variations and scenarios that were relevant to explain the effect of staying at home when having symptoms of one disease on the other, or the effect that twice as many symptomatic individuals stay at home for the most severe disease than for the other. Third, the model could be extended to more than two diseases or to other types of compartmental models such as the SEIR model (including a latent period) and the SIRS model (assuming a short period of immunity instead of life-long immunity). Fourth, we assumed that people stay at home at the onset of symptoms. In practice, people feel bad and stay home the day after. Fifth, competition between two pathogens was not taken into account. Competition could, among others, be included by assuming partial cross-immunity, or enhanced susceptibilty to one of the diseases compared to the other (Gao et al., 2016). Sixth, the model is a non-preferential model. This means that we assume that the infection risk is the same irrespective of whether a susceptible individual is contacting a symptomatic or asymptomatic individual. Moreover, asymptomatic and symptomatic cases recover at the same rate. The model can be extended to a preferential model, like described by Santermans et al. (2017) for mono-infections. Seventh, the model is a deterministic one, including age-specific contact patterns to describe age-related heterogeneity. The model assumes a constant distribution of contacts during the course of an epidemic. However, it has been reported that the average number of disease-causing contacts is higher at the start of an outbreak than in the end (Bansal et al., 2007). This means that our model may underestimate the number of infections at the start of the simulation and may overestimate them at the end. Heterogeneity in the distribution of contacts and other types of heterogeneity can be included into stochastic simulations (Britton, 2010). An interesting direction for future research is to extend the proposed model to a stochastic SIR model. Lastly, contact matrices for the 2009 A/H1N1pdm influenza from Van Kerckhove et al. (2013) were used. Using contact matrices for other strains or pathogens could influence our conclusions.

To our knowledge, this was the first study assessing the influence of changes in behavior on the joint dynamics of two infectious diseases. We can conclude that the reported effects are caused by different mixing patterns between asymptomatic and symptomatic individuals, and individuals staying at home. Furthermore, a take home message from this study is that assessing the joint dynamics of two or more infectious diseases is important to give advise on behavioral interventions. From a public health point of view, it is crucial to include age classes and differences in mixing patterns between symptomatic and asymptomatic cases in modeling studies.

## Appendix A Supplementary materials

Supplementary Data S1 Supplementary Figures. This is open data under the CC BY license http://creativecommons.org/licenses/by/4.0/ Supplementary Data S1
