# Supplementary material for: The impact of behavioral interventions on co-infection dynamics: An exploration of the effects of home isolation
Source: J Theor Biol. 2019 Sep 7;476:5–18. doi: 10.1016/j.jtbi.2019.05.017 (PMC6609929; doi:10.1016/j.jtbi.2019.05.017)
Supplement: Supplementary Data S1 — Supplementary Figures. This is open data under the CC BY license http://creativecommons.org/licenses/by/4.0/ [file mmc1.pdf]

Supplementary material for: The impact of behavioral interventions on co-infection dynamics: an exploration of the effects of home isolation

Diana M Hendrickx[a,*], Steven Abrams[a], Niel Hens[a,b]

[a] *Center for Statistics, Interuniversity Institute for Biostatistics and statistical Bioinformatics, Hasselt University, Diepenbeek, Belgium*
[b] *Centre for Health Economics Research and Modelling Infectious Diseases, Vaccine and Infectious Disease Institute, University of Antwerp, Antwerp, Belgium*

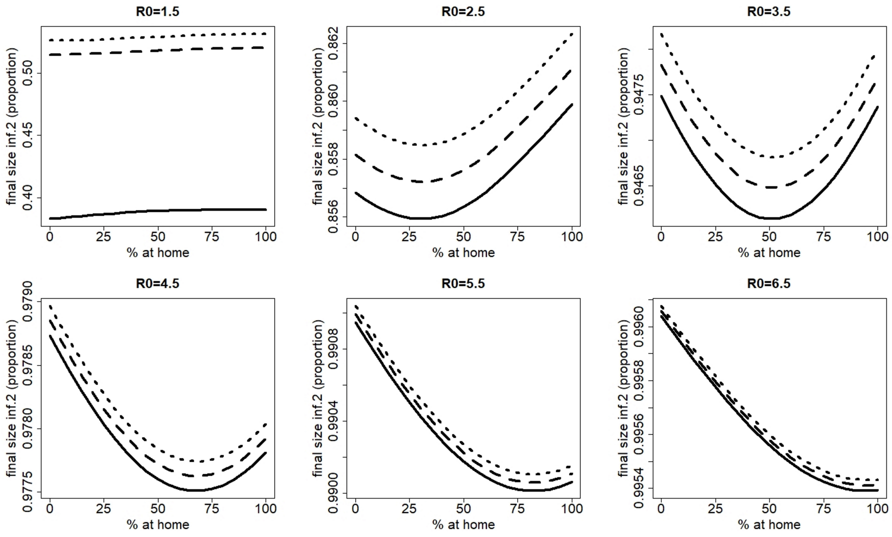

Figure S1: Influence of the infectious period on the behavior observed in Section 3.1. Infectious period of both infections is 7 days (dotted line); 14 days (dashed line); 21 days (solid line). All other parameters were taken from the first baseline scenario. $R_0 = R_{0,1} = R_{0,2}$.

*diana.hendrickx@uhasselt.be

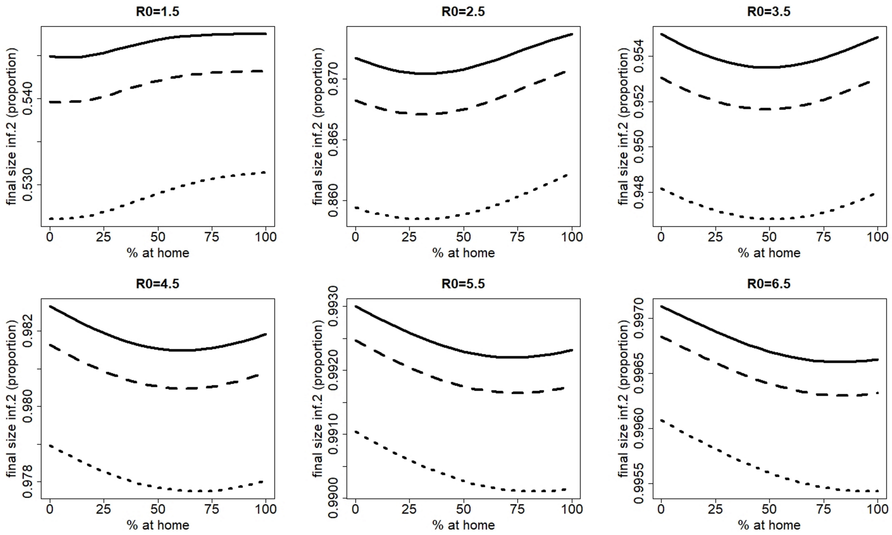

Figure S2: Influence of the infectiousness of symptomatic versus asymptomatic cases on the behavior observed in Section 3.1. Solid line: symptomatic cases are 9 times as infectious as asymptomatic cases for both infections; dashed line: symptomatic cases are 6 times as infectious as asymptomatic cases for both infections; dotted line: symptomatic cases are 3 times as infectious as asymptomatic cases for both infections. All other parameters were taken from the first baseline scenario. $R_0 = R_{0,1} = R_{0,2}$.

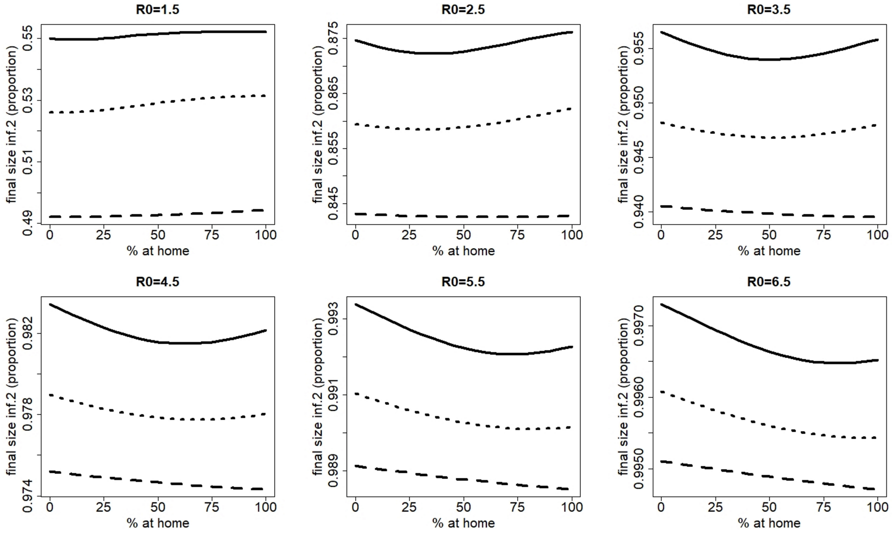

Figure S3: Influence of the fraction of symptomatic cases on the behavior observed in Section 3.1. Dashed line: 30% symptomatic for both diseases; dotted line: 60% symptomatic for both diseases; solid line: 90% symptomatic for both diseases. All other parameters were taken from the first baseline scenario. $R_0 = R_{0,1} = R_{0,2}$.

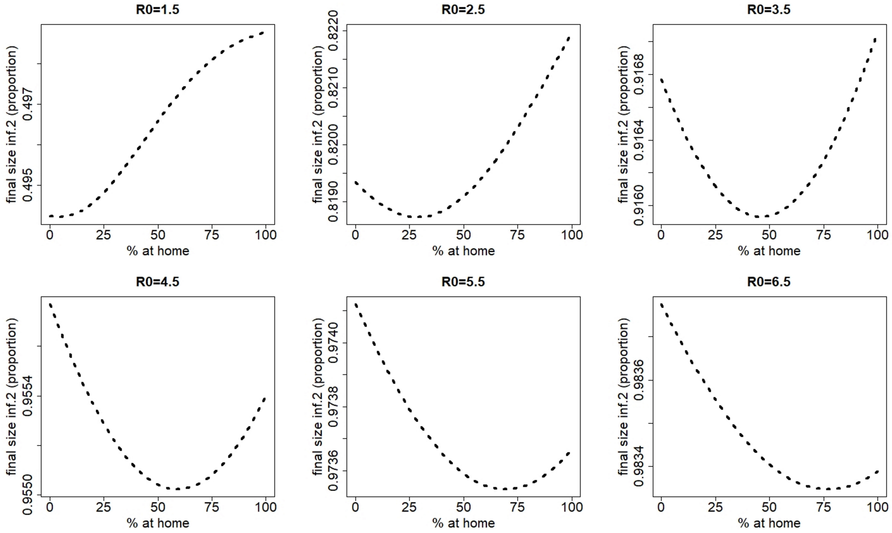

Figure S4: Final size of infection 2 (as a proportion of the total population) against the percentage staying at home when having symptoms of disease 1 for different values of $R_0 = R_{0,1} = R_{0,2}$ for a model with 86×86 contact matrices. The parameters used are those of the first baseline scenario.

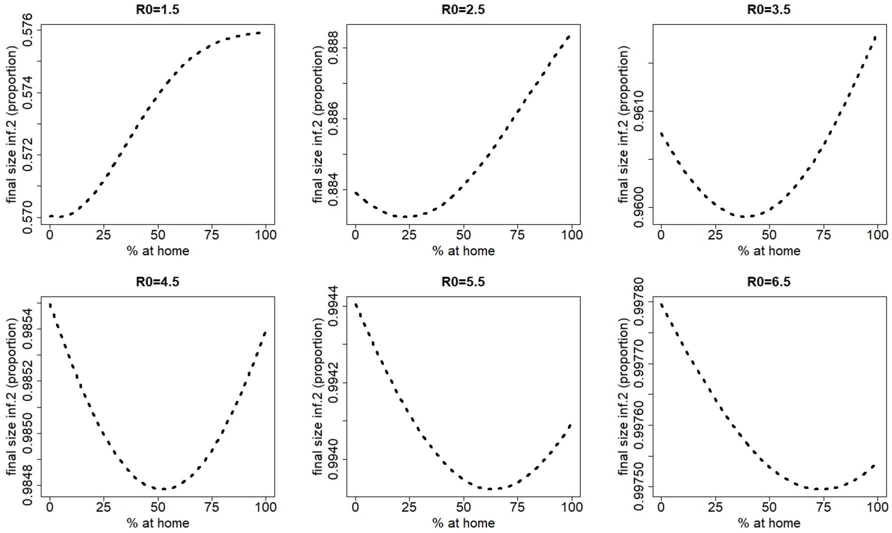

Figure S5: Final size of infection 2 (as a proportion of the total population) against the percentage staying at home when having symptoms of disease 1 for different values of $R_0 = R_{0,1} = R_{0,2}$ for a model with 1×1 contact matrices. The parameters used are those of the first baseline scenario.

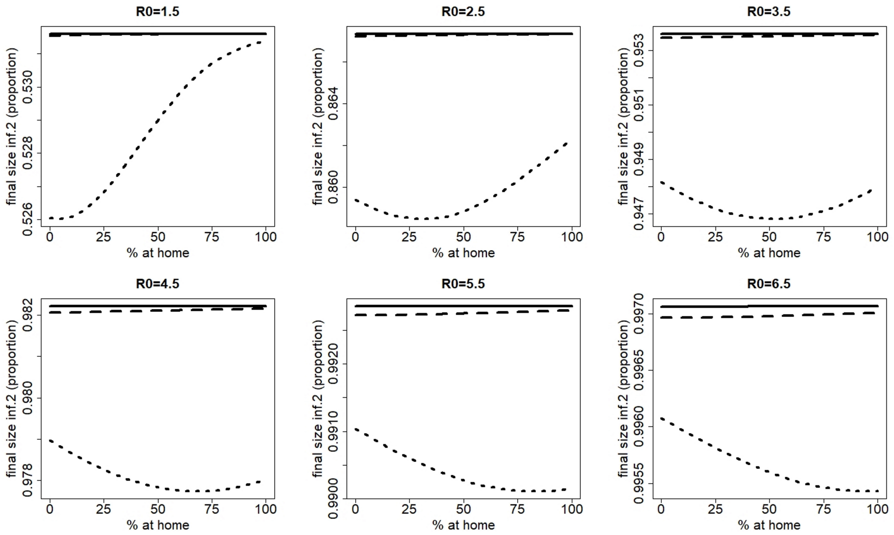

Figure S6: Influence of the infectious period of infection 1 on the behavior observed in Section 3.1. Infectious period for infection 2 is 7 days. Infectious period for infection 1 is 7 days (dotted line); 14 days (dashed line); 21 days (solid line). All other parameters were taken from the first baseline scenario. $R_0 = R_{0,1} = R_{0,2}$. We observe that the effect of staying at home for disease 1 on the final size of disease 2 is negligible if the infectious period of disease 1 is one or two weeks longer than the infectious period of infection 2.

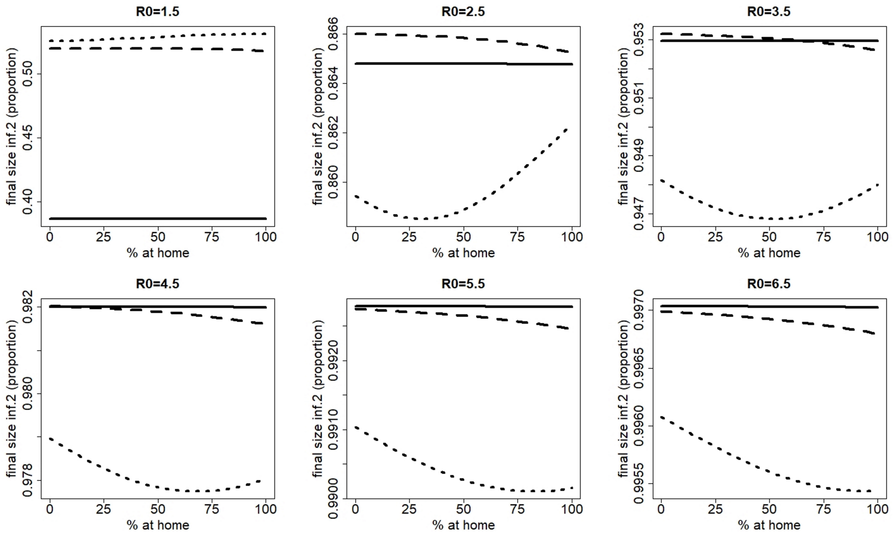

Figure S7: Influence of the infectious period of infection 2 on the behavior observed in Section 3.1. Infectious period for infection 1 is 7 days. Infectious period for infection 2 is 7 days (dotted line); 14 days (dashed line); 21 days (solid line). All other parameters were taken from the first baseline scenario. $R_0 = R_{0,1} = R_{0,2}$. We observe that the final size of infection 2 slightly decreases with an increasing percentage of people staying at home for disease 1 if the infectious period of disease 2 is 14 days (one week longer than the infectious period for infection 1). The effect of staying at home for disease 1 on the final size of disease 2 is negligible if the infectious period of disease 2 is 21 days (two weeks longer than the infectious period of infection 1).

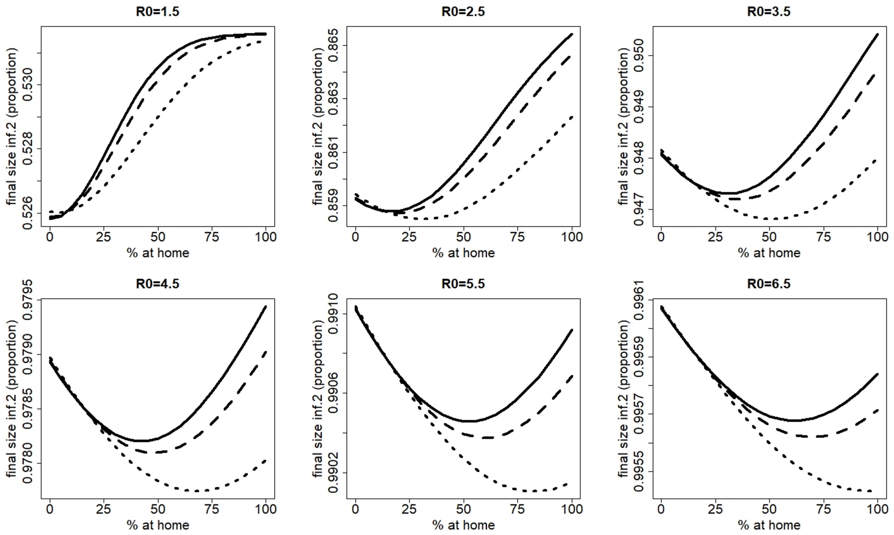

Figure S8: Influence of the infectiousness of symptomatic versus asymptomatic cases of infection 1 on the behavior observed in Section 3.1. For infection 2, symptomatic cases are 3 times as infectious as asymptomatic cases. Solid line: symptomatic cases are 9 times as infectious as asymptomatic cases for infection 1; dashed line: symptomatic cases are 6 times as infectious as asymptomatic cases for infection 1; dotted line: symptomatic cases are 3 times as infectious as asymptomatic cases for infection 1. All other parameters were taken from the first baseline scenario. $R_0 = R_{0,1} = R_{0,2}$. We observe that the infectiousness of symptomatic versus asymptomatic cases of infection 1 has little effect on the qualitative effects observed in section 3.1.

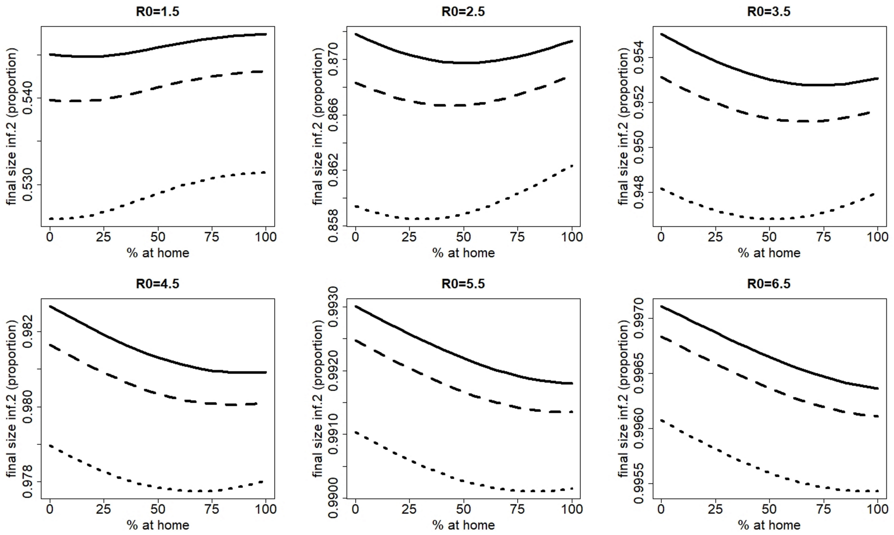

Figure S9: Influence of the infectiousness of symptomatic versus asymptomatic cases of infection 2 on the behavior observed in Section 3.1. For infection 1, symptomatic cases are 3 times as infectious as asymptomatic cases. Solid line: symptomatic cases are 9 times as infectious as asymptomatic cases for infection 2; dashed line: symptomatic cases are 6 times as infectious as asymptomatic cases for infection 2; dotted line: symptomatic cases are 3 times as infectious as asymptomatic cases for infection 2. All other parameters were taken from the first baseline scenario. $R_0 = R_{0,1} = R_{0,2}$. We observe that the infectiousness of symptomatic versus asymptomatic cases of infection 2 has little effect on the qualitative effects observed in section 3.1.

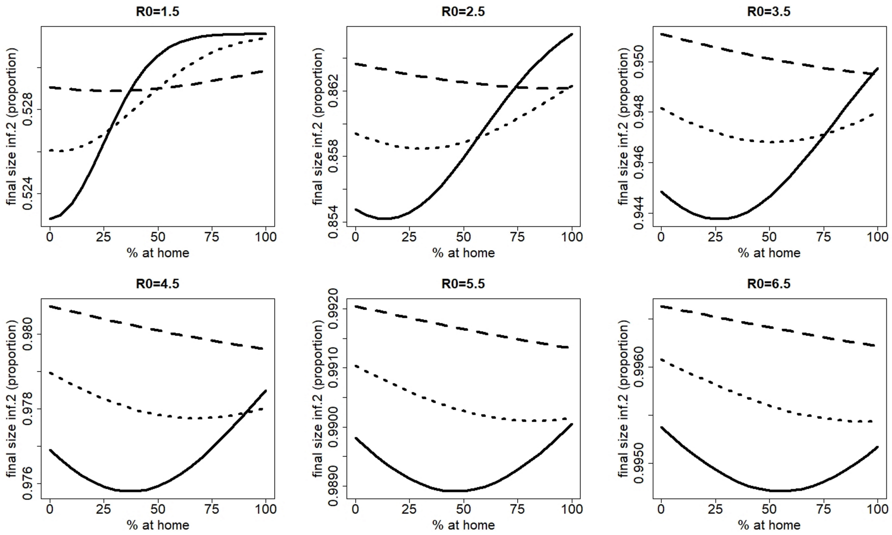

Figure S10: Influence of the fraction of symptomatic cases of disease 1 on the behavior observed in Section 3.1. Dashed line: 30% symptomatic for disease 1 and 60% symptomatic for disease 2; dotted line: 60% symptomatic for both diseases; solid line: 90% symptomatic for disease 1 and 60% symptomatic for disease 2. All other parameters were taken from the first baseline scenario. $R_0 = R_{0,1} = R_{0,2}$. We observe that the effects observed in Section 3.1 increase with an increasing percentage of symptomatic cases for disease 1.

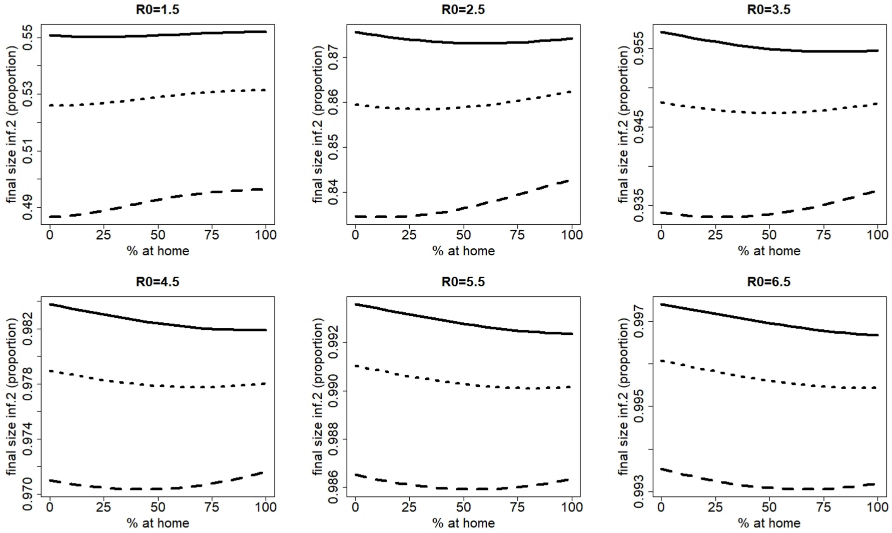

Figure S11: Influence of the fraction of symptomatic cases of disease 2 on the behavior observed in Section 3.1. Dashed line: 60% symptomatic for disease 1 and 30% symptomatic for disease 2; dotted line: 60% symptomatic for both diseases; solid line: 60% symptomatic for disease 1 and 90% symptomatic for disease 2. All other parameters were taken from the first baseline scenario. $R_0 = R_{0,1} = R_{0,2}$. We observe that the percentage of symptomatic cases of disease 2 has little effect on the qualitative effects observed in section 3.1.
